# Elevated Transcription of the Gene *QSOX1* Encoding Quiescin Q6 Sulfhydryl Oxidase 1 in Breast Cancer

**DOI:** 10.1371/journal.pone.0057327

**Published:** 2013-02-27

**Authors:** Mikhail Soloviev, Michelle P. Esteves, Fakhria Amiri, Mark R. Crompton, Christopher C. Rider

**Affiliations:** School of Biological Sciences, Centre for Biomedical Sciences, Royal Holloway University of London, London, United Kingdom; Sun Yat-sen University Medical School, China

## Abstract

The q arm of chromosome 1 is frequently amplified at the gene level in breast cancer. Since the significance of this is unclear we investigated whether 1q genes are overexpressed in this disease. The cDNA levels of 1q-located genes were analysed in a search for overexpressed genes. 26 genes mapping to the 1q arm show highly significant (P≤0.01) overexpression of transcripts in breast cancer compared to normal breast tissue. Amongst those showing the highest levels of overexpression in both expressed sequence tag (EST) and serial analysis of gene expression (SAGE) databases was enzyme quiescin Q6 sulfhydryl oxidase 1 (*QSOX1)*. We investigated *QSOX1* cDNA derived from T47D breast carcinoma cells by RT-PCR and 3′-RACE PCR and identified a novel extended form of *QSOX1* transcript, containing a long 3′UTR, nearly double the size of the previously reported *QSOX1* cDNA, and confirmed its 3′ end nucleotide sequence using RACE-PCR. We also used quantitative real-time PCR to analyse a panel of cDNAs derived from 50 clinically-graded normal and malignant breast tissue samples for the expression of *QSOX1* mRNAs. *QSOX1* transcription was elevated in an increasing proportion in the grade 2 and grade 3 tumours (graded according to the Nottingham prognostic index), with 10 of the 15 grade 3 tumours (67%) examined exceeding the normal range. There was a significant correlation between relative transcript level and clinical grade (P≤0.01) for all qPCR primer sets tested. *QSOX1* mRNA levels, based on SAGE expression data, did not correlate with either Estrogen Receptor (ER) or Epidermal Growth Factor Receptor 2 (ErbB-2 or HER2/neu) expression. Our data indicate that *QSOX1* is a potential new prognostic marker which may prove of use in the staging of breast tumours and the stratification of breast cancer patients.

## Introduction

It is well established that the chromosome 1q arm is subject to amplification at the DNA level in some 50–60% of breast cancers [1]–[3]. Although this is not the only chromosomal amplification in this disease, it is one of the largest and most common, and has been interpreted as representing an early event in breast carcinogenesis [1]. Estimates of the size and number of individual amplicons involved vary, but more recent high-resolution comparative genomic hybridisation studies have indicated that although the entire q arm can be amplified, the 1q21.1–1q31.1 and 1q32.1–1q44 regions are the most frequently affected [4], [5]. In contrast to 1q, the 1p arm shows little amplification, often having a loss of copy number [1], [3]–[5]. However, the functional significance of 1q gene amplification remains unknown. As might be expected, gene copy number is associated with mRNA overexpression in breast cancer tissue, but the correlation here is less than complete. A cDNA microarray study showed that 44% of genes highly amplified are strongly overexpressed in breast cancer, but so too are 6% of genes at normal copy number [6].

Since the whole 1q arm is subject to gene amplification, we hypothesised that multiple genes mapping in this genomic region are important in breast cancer tumourigenesis and/or progression. However to our present knowledge, only two 1q genes, *COX2* [7], [8] and peroxiredoxin-6*/PRDX6* [9] have thus far been found to be overexpressed in breast cancer and to have key roles in metastasis and cancer cell survival. A further 1q gene, nectin-4/*PVRL4*, is known to be overexpressed, but the pathological significance of this is as yet unclear [10]. In order to investigate whether further 1q genes might be overexpressed in breast cancer, we analysed their mRNA levels in normal and cancerous breast tissue using serial analysis of gene expression (SAGE) and EST data collected in the Cancer Genome Anatomy Project (http://cgap.nci.nih.gov). The results of these analyses reveal that several genes show particularly high levels of overexpression including *QSOX1* (NM_002826) which encodes the enzyme quiescin Q6 sulfhydryl oxidase 1. This enzyme belongs to a family of flavin adeninedinucleotide (FAD) - dependent sulfhydryl oxidases [11].

We confirmed our bioinformatics findings for *QSOX1* experimentally, by conducting RT-PCR, 3′RACE PCR and quantitative real-time PCR. We identified a novel extended 3′UTR form of the *QSOX1* transcript and showed that the gene is indeed overexpressed in breast cancers of poor prognosis. Our data identify *QSOX1* as a gene worthy of further detailed investigation to define its relevance in the pathogenesis and progression of this disease.

## Results

### Overexpression and 3′ Extension of QSOX 1 Transcripts in Breast Cancer

In order to investigate whether the frequent gene amplification of the q arm of chromosome 1 might be associated with overexpression of the genes located in this region, we investigated a pair of matching SAGE libraries using the DGED tool. The SAGE data showed that 156 1q arm genes undergo transcriptional upregulation in breast ductal carcinoma compared to normal breast tissue. The upregulation of approximately one third of these genes was considered significant (P≤0.05) and 25 of these genes show highly significant upregulation (P≤0.01). The latter upregulated genes are listed in Table 1 in order of highest degree of their overexpression in breast cancer; genes with (0.01<P≤0.05) are listed in the Supplementary Table S1. The expression of four genes shown was not detectable in the library derived from normal tissue (Table 1), resulting in an ‘infinite-fold increase’ in cancer tissue. All the entries listed were also investigated for their expression in EST libraries. However, because of the limited EST data available no quantitative analysis was possible, EST data were therefore considered as only qualitative. Therefore in Table 1 EST expression results are shown solely as the presence or absence of the relevant cDNA in the two pools of cDNA libraries. Of the four highest ranked genes, which had no detectable expression in normal breast tissue in the SAGE database (with P<0.01), we chose to focus on *QSOX1* which encodes quiescin Q6 sulfhydryl oxidase 1, which has not been previously associated with breast cancer. *QSOX1* was found to be overexpressed in breast cancer on mRNA level in SAGE expression library, but expressed below the detection level in the matched normal SAGE library (P = 0.01) and was also detected in breast cancer tissue but not normal breast in the EST database.

**Table 1:** Upregulated expression of 1q genes in breast ductal carcinoma.

Gene Symbol^1^	Gene Name	Chromosome position^2^	SAGE^3^ cancer (A)	SAGE^3^ normal (B)	Normalised Odds A:B^4^	EST^5^ cancer (A)	EST^6^ normal (B)
UBAP2L	Ubiquitin associated protein 2-like	**1q21.3**	12	0	∞	**−**	**−**
QSOX1	Quiescin Q6 sulfhydryl oxidase 1	**1q24**	10	0	∞	**+**	**−**
PVRL4	Poliovirus receptor-related 4	**1q22–q23.2**	9	0	∞	**−**	**−**
ACBD3	Acyl-Coenzyme A binding domain containing 3	**1q42.12**	9	0	∞	**−**	**−**
GLUL	Glutamate-ammonia ligase (glutamine synthetase)	**1q31**	29	1	16.27	**+**	**−**
CCT3	Chaperonin containing TCP1, subunit 3 (gamma)	**1q23**	14	1	7.85	**+**	**−**
SLC30A1	Solute carrier family 30 (zinc transporter), member 1	**1q32–q41**	13	1	7.29	**−**	**−**
UCHL5	Ubiquitin carboxyl-terminal hydrolase L5	**1q32**	37	3	6.92	**−**	**−**
PI4KB	Phosphatidylinositol 4-kinase, catalytic, beta	**1q21**	11	1	6.17	**−**	**−**
GPATCH4	G patch domain containing 4	**1q22**	11	1	6.17	**+**	**−**
TMCO1	Transmembrane and coiled-coil domains 1	**1q22–q25**	16	2	4.49	**+**	**−**
GUK1	Guanylate kinase 1	**1q32–q41**	23	3	4.30	**+**	**−**
MAPKAPK2	Mitogen-activated protein kinase-activated protein kinase 2	**1q32**	28	5	3.14	**−**	**−**
S100A14	S100 calcium binding protein A14	**1q21.3**	89	16	3.12	**−**	**−**
RPS6KC1	Ribosomal protein S6 kinase, 52 kDa, polypeptide 1	**1q41**	20	4	2.81	**−**	**−**
ZNF669	Zinc finger protein 669	**1q44**	44	10	2.47	**−**	**−**
CRABP2	Cellular retinoic acid binding protein 2	**1q21.3**	28	7	2.24	**+**	**−**
HAX1	HCLS1 associated protein X-1	**1q21.3**	21	6	1.96	**−**	**−**
PSMD4	Proteasome (prosome, macropain) 26S subunit, non-ATPase, 4	**1q21.3**	17	5	1.91	**−**	**−**
ELF3	E74-like factor 3 (ets domain transcription factor, epithelial-specific)	**1q32.2**	10	3	1.87	**−**	**−**
H3F3A	H3 histone, family 3A	**1q41**	57	18	1.78	**−**	**−**
ENSA	Endosulfine alpha	**1q21.3**	22	7	1.76	**+**	**−**
LMNA	Lamin A/C	**1q21.2–q21.3**	132	43	1.72	**+**	**−**
TAGLN2	Transgelin 2	**1q21–q25**	64	21	1.71	**+**	**−**
F11R	F11 receptor)	**1q21.2–q21.3**	142	51	1.56	**+**	**+**

1 The genes are listed in the order of degree of overexpression in cancer tissue. For all genes listed, the significance factor is P≤0.01.

2 From Unigene (http://www.ncbi.nlm.nih.gov/UniGene).

3 Total number of short SAGE tags identified for each individual gene.

4 The sequences Odds ratio is obtained by calculating normalised values of expression of each gene (total number of SAGE tags divided by the total number of tags in each library: 66,128 tags in the breast ductal carcinoma library and 50,512 tags in normal epithelium), and then calculating the ratio of these values (normalised expression in cancer over normalised expression in normal tissue). For the four top entries gene expression was not detected in normal tissues (SAGE only).

5 Based on ten non-normalised cDNA EST libraries from cancer breast tissues (not cell lines), totalling 11,161 sequences available.

6 Based on two non-normalised cDNA EST libraries from normal breast tissues (not cell lines), totalling 1,485 sequences available.

**Figure 1:**
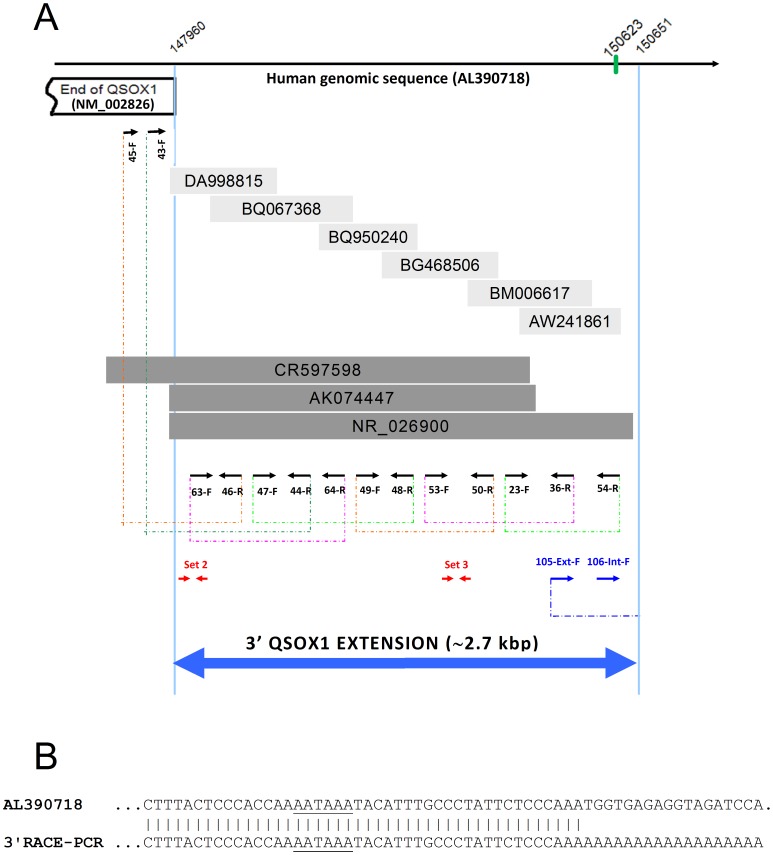
3′ Extension of *QSOX1* cDNA. Panel A: Overlapping database sequences are aligned with the fragment of human genomic sequence from chromosome **1** (AL390718). The 3′ end of *QSOX1* exon 12 (NM_002826) is shown as an open box. Light grey shaded boxes show selected overlapping ESTs (accession numbers are indicated). Dark grey boxes denote the overlapping cDNAs identified in GenBank (accession numbers are indicated). Black arrows indicate the approximate positions and orientation of the PCR primers used to check the expression of the extended *QSOX1* transcript. Primer names are shown next to their positions. Dashed lines show the individual overlapping PCR products obtained, which continuously cover the *QSOX1* 3′ extension. Blue arrows indicate the approximate positions of the sequence-specific RACE-PCR primers used (see Supplementary Table S2 for all the primers’ sequences). The polyadenylation site (AATAAA) was found approximately 30 bases upstream of the poly(A) tail (positions 150623 and 150651 respectively). All positions are numbered relative to the human genomic sequence (AL390718). Red arrow sets indicate the approximate positions of real time qPCR primers sets 2 and 3. The blue double-headed arrow indicates the experimentally confirmed 3′ extension of *QSOX1* cDNA. Panel B: Alignment of the experimentally identified 3′ end of the *QSOX1* cDNA with the human genomic sequence (AL390718). The polyadenylation signal (AATAAA) is underlined in both sequences.

**Figure 2:**
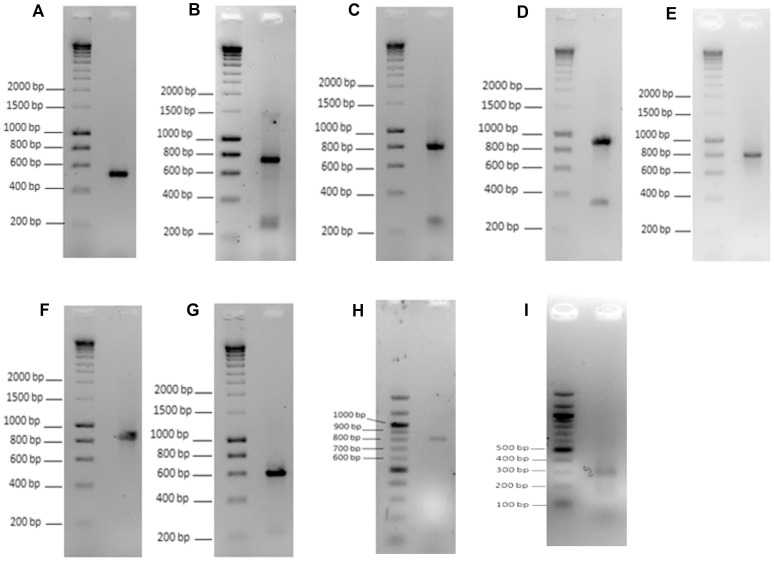
RT-PCR and RACE-PCR amplification of the extended form of the *QSOX1* cDNA. Panel **A** shows amplified cDNA using primers 45-F and 46-R (expected length 550 bp). Panel **B** shows amplified cDNA using primers 43-F and 44-R (expected length 735 bp). Other PCR amplifications were using primers: 63-F with 64-R (panel **C,** expected size 836 bp), 47-F with 48-R (panel **D,** expected size 922 bp), 49-F with 50-R (panel **E,** 825 bp), 53-F with 36-R (panel **F,** 883 bp), 23-F with 54-R (panel **G,** 620 bp). Panel **H** shows amplified cDNA of the soluble version of *QSOX1* (*QSOX1*b) using primers 17-F and 18-R (expected length 814 bp). Panel **I** shows the size of the cDNA fragment amplified with the specific sense primer (106-Int-F) and antisense 3′ RACE adapter primer (Adap-1-R). The specificity of amplification (the identity of all amplified PCR products) was further confirmed by DNA sequencing for all amplified products.

Whilst analysing the extent of EST coverage of *QSOX1* cDNA we identified an EST fragment (DA998815) overlapping with the most 3′ end of the reported *QSOX1* cDNA (NM_002826) by 14 nucleotides and extending for more than 500 bases beyond the established 3′ end of NM_002826. We therefore used a fragment of genomic sequence immediately beyond the 3′ end of *QSOX1* to search human sequence databases for other possible matches. We identified a region of approximately 7,000 bp downstream of *QSOX1* containing many ESTs and a few longer cDNA sequences of which some provided a continuous overlapping region of over 2.5 kbp long. A selection of these is shown in Figure 1A, as a series of overlapping sequences indicating a potentially substantial 3′ extension of the *QSOX1* cDNA. To prove the existence of the predicted extended version of *QSOX1* cDNA experimentally we designed a set of PCR primers (detailed in the Supplementary Table S2) with the aim of obtaining a series of overlapping RT-PCR products at the 3′ end of the *QSOX1* gene. Using cDNA from breast cancer derived T47D carcinoma cells we were able to amplify cDNA fragments and to achieve continuous sequence coverage starting from the position 147692 (middle of exon 12 of *QSOX1*) to position 150620 of the genomic DNA (AL390718) (Figure 1A). Individual amplified cDNA fragments are shown in Figure 2, panels A–H. All of the fragments were of the expected length and all were confirmed by sequencing.

In order to identify the actual 3′ end of the extended *QSOX1* cDNA, we carried out 3′-RACE-PCR using a set of nested specific primers and a set of 3′-RACE adapter primers (see Supplementary Table S2 for sequences). The amplified cDNA was detected as a ∼300 bp band, see Figure 2, panel I, and the 3′-end of the *QSOX1* cDNA was confirmed by sequencing. The end of the extended *QSOX1* cDNA corresponds to position 150651 of the genomic sequence (AL390718**),** see Figure 1B. We also identified a typical polyadenylation signal AATAAA positioned approximately thirty nucleotides upstream of the poly-A sequence in the cDNA (Figure 1B). The combination of the overlapping RT-PCRs, of the 3′-RACE-PCR and the polyadenylation signal confirmed unequivocally the position of the true 3′ end of the *QSOX1* cDNA. Since the translation of *QSOX1* stops inside exon12 which contains a stop codon, the newly identified extension is therefore a long non-coding 3′UTR.

### Quantitative Real-time PCR of *QSOX1* Transcripts in Breast Cancer

**Figure 3:**
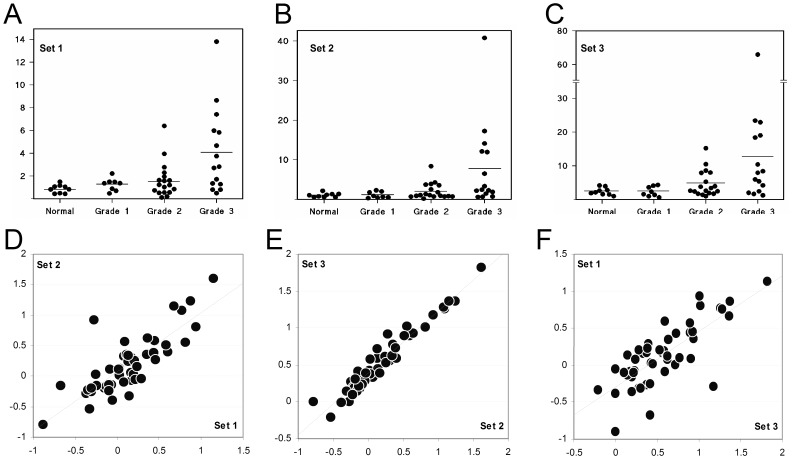
Quantitative Real-Time PCR of *QSOX1* transcripts in a panel of RNA samples from normal and breast cancer tissue samples, clinically graded using the Nottingham prognostic index. Data for three different TaqMan probe sets are shown (panels A-C). In all panels the vertical axis is relevant abundance normalised to 18s rRNA levels. A: Primer Set 1 (based on the *QSOX1* CDS). B: Primer Set 2 (based on the *QSOX1* 3′UTR). C: Primer Set 3 (based on the *QSOX1* 3′UTR). The horizontal line in each dot plots shows mean expression data for each probe set/condition D: Scatter plot for Set 2 vs. Set 1 data across all preparations. E: Scatter plot for Set 3 vs. Set 2 data across all preparations. F: Scatter plot for Set 1 vs. Set 3 data across all preparations. For all scatter plots, all axes show relevant abundance values for the relevant probe set in logarithmic scale. Best fit linear regression is shown as a dotted diagonal line.

We further sought to confirm experimentally both the existence of this new 3′ *QSOX1* mRNA extension and the upregulation of *QSOX1* mRNA levels in breast cancer tissue. Hence real-time PCR was conducted using sets of probes based on the *QSOX1* coding sequence (CDS) (Set 1) and on the newly identified 3′UTR extension (Sets 2 and 3, see Figure 1A). A panel of cDNA samples prepared from surgically removed breast tissue was screened. All tissues tested revealed the transcription of all the three *QSOX1* regions tested, confirming that the *QSOX1* mRNA exists as a ∼6 kbp transcript, much longer than previously thought. The expression of all of the three regions was low in normal tissues and in grade 1 tumours, but showed elevated levels in an increasing proportion of the grade 2 and grade 3 tumours, Figure 3A–C. Non-parametric statistical analysis across all four clinical classifications showed a Spearman’s correlation coefficient of 0.53 between relative transcript level and clinical grade, a value significant at the *p* 0.01 level. Of the 15 grade 3 tumours examined, expression in 10 samples exceeded the upper limit of normal (mean +2 standard deviations) for each primer set.

Of the 41 patients whose cDNA preparations were subject to real time PCR analysis, 25 had tumours classified as ductal (mean relative transcript level, 3.21) and 9 were lobular (mean value, 1.346). The remaining 7 patients were distributed between the mixed (4), mucinous (2) and cribriform (1) pathological classifications. For the results obtained with the Set 1, shown in Fig. 2A, a Two-tailed t tests established a significance difference between the normal mean and the ductal tumour mean (p = 0.00148), and between the ductal and lobular mean values (p = 0.120). There was however no significant differences in the mean values for normal tissue and the lobular tumours (p = 0.112).

**Figure 4:**
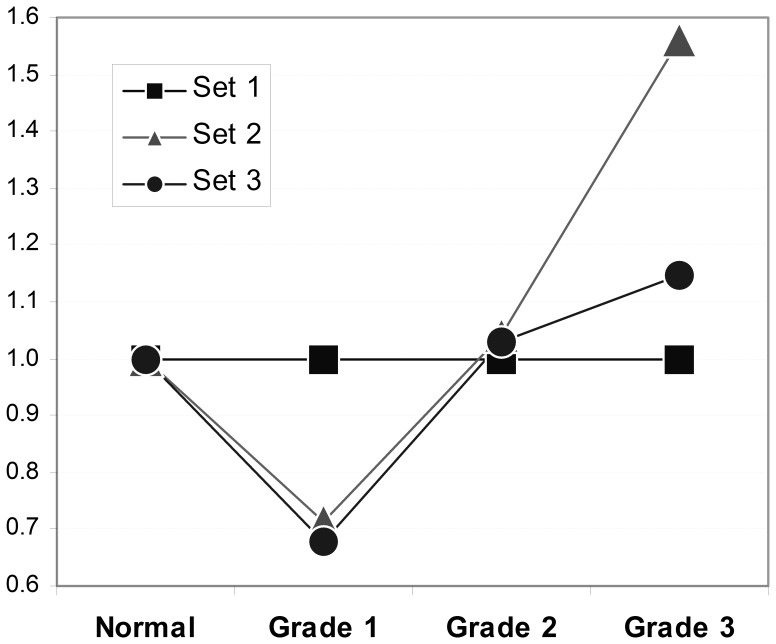
Differential overexpression of the CDS and 3′UTR of *QSOX1*. Mean expression data (as in Figure 2A–C) for the three different TaqMan probe sets normalised per mean expression data for probe Set 1 are shown. Sets 2 and 3 (3′UTR region of *QSOX1*) show a lower degree of expression in Grade 1 tumours and a higher degree of expression in Grade 3 tumours relative to Set 1 (*QSOX1* CDS).

Correlation of the expression levels of individual regions (probe sets 1, 2 and 3) in individual tissue samples, showed some differences in degree of scattering between signals measured with the Set 1 probe (*QSOX1* CDS) and either of the Set 2 or 3 probes, both of which are within the newly identified extended *QSOX1* 3′UTR, (see Figure 3D–F). This raise further the possibility that alternative splicing events might occur to account for a degree of variability in the expression of distinct regions of the transcript. When normalised against expression in normal breast tissue the data revealed that the *QSOX1* CDS expresses at relatively higher levels in Grade 1 cancers, compared to the expression level of the *QSOX1* long 3′UTR (both Set 2 and Set 3 probes), but the trend is reversed in Grade 2 and 3 cancers (Figure 4).

We attempted to identify experimentally any alternatively spliced *QSOX1* transcripts which could have contributed to the above observations by PCR amplification of cDNA derived from T47D carcinoma cells using primer pairs designed around all known and predicted splice variants. We did not detect any further alternatively spliced variants beyond the previously reported *QSOX1*a and *QSOX1*b forms (the latter encoding the soluble form of the protein [12] and the extended 3′-UTR reported above.

**Figure 5:**
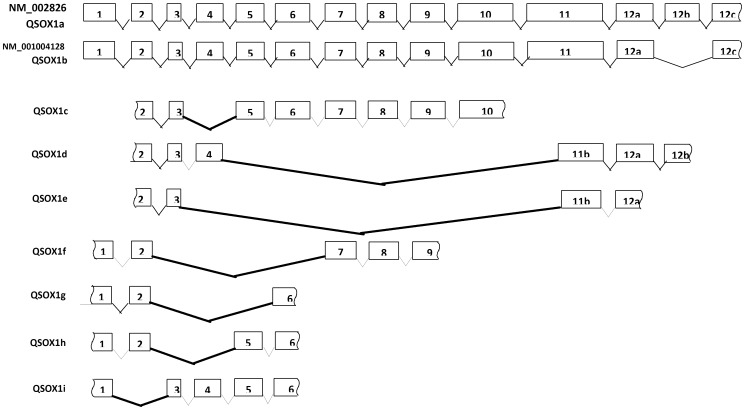
Alternative splicing of the *QSOX1* mRNA. *QSOX1* exons are shown. Alternatively spliced variants *QSOX1*a and *QSOX1*b are based on NM_002826 and NM_001004128 respectively. Other alternative splicing variants are based on the EST mapping data (see Table 2) and are named *QSOX1*c – *QSOX1*i for consistency with the previously used nomenclature. The identified splicing sites are shown with bold connecting lines. Exon 12b denotes the sequence fragment missing in the shorter alternatively spliced version of *QSOX1*b.

**Figure 6:**
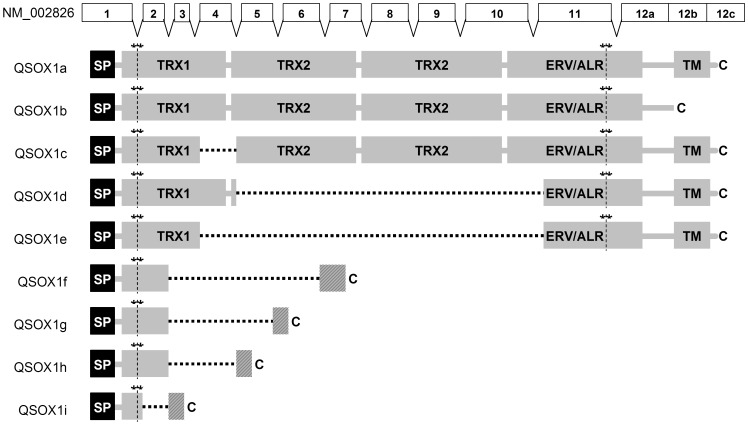
*QSOX1* internal splicing variants and their translation. *QSOX1* protein domains Trx1, Trx2, HRR and ARV/ALR are shown as light grey shaded boxes. Asterisks and vertical yellow lines indicate the position of cysteine-pairs in the product of the *QSOX1* transcript. SP (black boxes) denotes a signal peptide, TM indicates a transmembrane domain and C indicates a C terminus. Square dotted horizontal lines indicate the joining of truncated domains. Hatched boxes show amino acid sequences produced where such joining results in reading frame-shifts.

We therefore searched EST databases for any evidence of additional alternative splicing. We identified eight ESTs showing novel putative alternative splicing patterns for the *QSOX1* mRNA. These are summarised in Figure 5 and Table 2. Since these sequences show alternative splicing, all of which occurs at the existing intron-exon boundaries, and they do not have any unspliced intronic sequences, it is likely that these may indeed represent alternative splicing rather than random cloning artefacts, despite them being undetectable in T47D carcinoma cells. The two putative alternative splicing variants which could have contributed to the differences in expression level between exon 7 and the 3′UTR would be *QSOX1*d and *QSOX1*e (Figure 5). It is interesting that in the protein products of both of these splice variants all three CxxC disulfide motifs present in the full length *QSOX1* and essential for the activity of the protein [12] remain intact. In *QSOX1*d the Thioredoxin TRX1 domain remains intact, whilst the TRX2 and HRR domains are removed completely, making these isoforms similar to plant QSOXs which lack the second Trx domain but have the same three redox sites. TRX1 is truncated at its C terminus in *QSOX1*e and both of the splicing isoforms encode slightly N-terminally shortened ERV/ALR domains. Both splicing variants preserve the *QSOX1* reading frame. Protein domain structures for the products of these and the other putative *QSOX1* alternative splice variants are summarised in Figure 6.

**Table 2:** Putative new QSOX1 splicing variants and their corresponding ESTs.

Splicing Variants	Corresponding ESTs
QSOX1**c**	BI913393
QSOX1**d**	BP341971
QSOX1**e**	BG978499
QSOX1**f**	BQ930111
QSOX1**g**	BX334202
QSOX1**h**	DA785209
QSOX1**i**	DB283802

## Discussion

Our bioinformatics analyses clearly indicate that a number of the genes located in sub-regions of the q arm of chromosome 1 which are commonly amplified at the DNA level in breast cancer [1]–[5] are also highly significantly upregulated at the mRNA level compared to normal breast tissue (Table 1). For example the *PVRL-4* gene product, Nectin-4 was found to be undetectable in normal breast epithelium, but expressed in some 60% of ductal breast carcinomas, correlating strongly with basal-like markers [10]. Another gene, *GLUL* encodes the enzyme glutamine synthetase which is highly expressed both in normal breast luminal epithelial cells and in luminal breast cancer [13]. Both mitogen-activated protein kinase-activated protein kinase 2 protein levels and enzyme activity are elevated in breast cancer [14]. *S100A14* overexpression is also well established in breast cancer. Thus Leth-Larsen et al. [15] derived a single cell clone of a human primary ductal breast carcinoma cell line HMT309 with an epithelial-line cancer stem phenotype and established that it expressed higher levels of *S100A14* protein than the parental cell line. Elevated levels of the *ELF3* protein have been detected in some but not all breast cancer cell lines [16]. Transgelin 2 was amongst a number of proteins found by LC-MS analysis to be overexpressed in microvessels isolated from invasive ductal breast carcinoma compared to those from adjacent non-malignant tissue [17].

Of particular interest was the identification among the found genes, of *QSOX1*, which encodes quiescin Q6 sulfhydryl oxidase 1, because no information existed at the time of its association with breast cancer. We confirmed our findings independently using bioinformatics approach and experimentally by RT-PCR, 3′RACE PCR and quantitative real-time PCR. We demonstrate here that the transcript for *QSOX1* is highly overexpressed in some breast cancers, with a strong correlation between expression level and prognostic index. Indeed, out of 15 cases with the worst prognostic index, 10 (67%) showed expression levels above the normal range. This high rate of overexpression is at least comparable with that of established prognostic markers such as *ESR1* or *HER2* [18]. However, the expression pattern of *QSOX1* correlated with neither *ESR1* nor *HER2* (Supplementary Figure S1), indicating that quiescin Q6 might be a new prognostic factor suitable for cancer staging and for the further stratification of patients with breast cancer. In this respect overexpression of *QSOX1* may have good prognostic potential and would be technically more straightforward to test than, for example, the reduction in the expression level of stromal caveolin *Cav-1*, reported recently to be indicative of advanced breast cancer, metastases, early disease recurrence and poor outcome [19], [20]. Our results show that the *QSOX1* transcript was significantly elevated in ductal tumours, but since this classification also included all tumours of advanced grade, the patient sample set we studied does not enable us to disentangle the effects of tumour classification versus tumour grade.

Overexpression of *QSOX1* in breast cancers has not previously been reported, however in a recent proteomics study, quiescin Q6-derived peptides were found to be secreted at elevated levels by two breast cancer derived cell lines, BT474 and MDA-MB-468, compared to the normal breast epithelial cell line, MCF-10A [21]. Transcription of *QSOX1* in oestrogen receptor-expressing breast cancer cell lines has also been documented in two further studies, although in one of these expression was found to be repressed by 17β-oestradiol [22] whereas in the other such treatment resulted in a modest induction [23]. In other cancers, peptides derived from a C-terminal part of the secreted form of *QSOX1* have been identified by liquid-chromatography-tandem mass spectrometry (LC-MS/MS) in the plasma of patients with ductal adenocarcinoma of the pancreas, but not in normal healthy donors, leading to the suggestion of *QSOX1* being a biomarker of cancer of the pancreas [24]. Moreover in a rodent model of prostate cancer, *QSOX1* is highly overexpressed in prostatic hyperplasia and intraepithelial neoplasia (PIN) lesions of mice lacking the transcriptional regulator and tumour suppressor gene *Nkx3.1* [25]. The overexpression of *QSOX1* in different types of tumours, and the ability to detect the protein or its fragments in body fluids including plasma [24], and urine [26], [27] makes *QSOX1* a promising target for studying as a candidate universal prognostic cancer marker gene.

Previously, only two alternative splicing-derived variants of the enzyme had been reported in human, mouse and rat tissues (NM_002826 and NM_001004128 human sequences encoding short and long mRNAs respectively). The latter encodes a full length membrane anchored protein, found in the Golgi, secretory granules, and in the endoplasmic reticulum [28], [29]. The shorter version of the *QSOX1* mRNA is produced by an alternative splicing event, whereby a 733 base long fragment is deleted from the middle of exon 12 resulting in a truncated *QSOX1* protein missing the transmembrane domain, and this encodes the secreted isoform [12], [30]. The extended 3′UTR reported here does not seem to encode an alternative protein sequence. Most likely it is involved in the post-transcriptional regulation of *QSOX1* mRNA translation and mRNA nuclear export, cleavage and polyadenylation. 3′UTRs are considered vital for transcript stability and gene expression regulations such as translational repression mediated by RNA-binding proteins and micro RNAs [31]–[33]. The *QSOX1* 3′UTR extension reported here could be involved in the regulation of *QSOX1* transcript stability and may explain the observed variability in the apparent levels of *QSOX1* CDS vs. 3′UTR regions.

Our systematic analysis of EST databases revealed eight putative new *QSOX1* splice variants. Of these three sequence variants, *QSOX1*c, d and e, have an internal in-frame deletion which preserves fully or partially the primary structure of the two main known functional domains of the protein: the PDI-like oxidoreductase thioredoxin domain Trx1 and the FAD - dependent sulfhydryl oxidase domain ERV/ALR [34]–[37]. The thioredoxin domain, Trx2, and the helix-rich-helix region HRR are deleted from all but the *QSOX1*c variant. The retaining of the main functional domains of the protein and all of the redox-active disulfides in the isoforms *QSOX1*c,d,e may preserve the main function of the enzyme - the oxidation of sulfhydryl groups to disulfides by reducing oxygen to hydrogen peroxide. This assumption is in agreement with the recently published functional studies of *QSOX1* fragments which indicated the importance of interaction of the Trx and ERV/ALR domains for the thiol-oxidation activity of the *QSOX1* protein [37], [38]. The products of the remaining splice variants (*QSOX1*f-i) lack all but the N-terminal fragment of the Trx1 domain and are most likely dysfunctional proteins.

This enzyme belongs to a family of flavin adeninedinucleotide (FAD) - dependent sulfhydryl oxidases [11]. It is a multi-domain protein formed by fusion of two ancient genes. The N-terminal region has a tandem pair of thioredoxin (TRX) domains, related to protein disulfide isomerase (PDI), and there is a C-terminal sulfhydryl oxidase–like endogenous retroviral element (ERV1). It also has a helix-rich-helix region (HRR) domain and a transmembrane domain. This enzyme catalyses the oxidation of protein thiol groups to disulphides with attendant reduction of oxygen to hydrogen peroxide [12], but its cellular role is currently unclear. Although initially identified as being strongly up-regulated in fibroblasts reaching confluence, it has also been since associated with growth factor activity, and it is highly expressed in cells with a large secretory load [12]. In different studies on *QSOX1* a variety of functions have been ascribed to this enzyme including protection from apoptosis [11], [12], [39]–[42] and facilitation of tumour cells invasion [38]. Thus, the forced overexpression of quiescin 6 in human MCF-7 breast cancer cells rendered them more resistant to apoptosis arising from oxidative stress compared to control transfected cells, thereby implicating *QSOX1* in cell survival [11] whilst the suppressed expression of quiescin in pancreatic cancer cell lines BxPC-3 and Panc-1 inhibited cancer cell invasion by activation of MMP-2 and MMP-9 Matrix Metalloproteinases [43]. The consequences of elevated levels of quiescin Q6 mRNA and its splicing variants in breast cancer are currently far from certain.

Quiescin Q6 is found both within the endoplasmic reticulum/Golgi apparatus and as a secreted protein [12]. This dual location, together with its activity towards unfolded polypeptides implies functions both in the initial folding of secreted proteins and their remodelling once they have reached the cell surface and extracellular matrix. The latter activity might be related to cancer cell signalling, migration and metastasis. In this context it is pertinent that in a recent genetic screen of cDNAs searching for transcripts able to promote the metastasis of the breast cancer cell line 168FARN, the thiol isomerase ERp5 was identified as promoting tumour cell migration and invasion, and as being up-regulated in invasive clinical breast cancer samples [44]. Taken together with our findings, it is emerging that the cellular mechanisms for the formation of appropriate disulphide linkages in secreted proteins is a fertile area of investigation in breast cancer of poor prognosis.

## Methods

### SAGE and EST Expression Data Analysis

cDNA and SAGE databases accessible on-line through the Cancer Genome Anatomy Project (CGAP) portal (http://cgap.nci.nih.gov) were used for the initial analysis of the expression of chromosome 1 genes in normal and cancerous breast tissues. The SAGE experimental viewer (SEV) and digital gene expression displayer (DGED) (http://cgap.nci.nih.gov/Tissues/SAGE) were used to identify genes potentially overexpressed in breast cancer. We used a matched pair of libraries derived from breast ductal carcinoma (BEREP4+_AP_DCIS_2, containing 66128 short SAGE tags) and normal epithelium (BEREP4+_AP_N2, containing 50512 short SAGE tags), both libraries were derived from tissues obtained from the same patient. Both under- and over- expressed genes were selected in SAGE DGED (F = 1) and no limitations were put on the values of the significance factor (P = 1). DGED analysis yielded 1915 SAGE tags (data not shown), Because some of the genes were represented by more than one short SAGE sequence tag, all such entries were combined. Unique gene entries having the significance factor P<0.2 were then annotated to their respective chromosome arms and cytogenetic bands and those genes located on the “q” arm of chromosome 1 were selected for analysis.

To confirm the results of the SAGE DGED search independently, we used expressed sequence tag (EST) expression data and the DGED tool (http://cgap.nci.nih.gov/Tissues/GXS). The following criteria were used to select libraries. For pool A (“cancer”): tissue selection - mammary gland/breast; minimum number of sequences per library - 10; library protocol - non-normalised, tissue histology - cancer. The same settings were used for pool B (“normal”), except for the tissue histology, which was set to normal. For the both pools, only human EST libraries were selected, all types were allowed (CGAP, MGC and ORESTES); in neither case were libraries from cell lines used. These settings yielded 10 cancer libraries (NCI_CGAP_Br1.1, NCI_CGAP_Br12, NCI_CGAP_Br22, NCI_CGAP_Br16, NCI_CGAP_Br18, NCI_CGAP_Br15, NCI_CGAP_Br3, NCI_CGAP_Br13, NCI_CGAP_Br17 and NIH_MGC_151) and two normal libraries (NCI_CGAP_Br14 and NCI_CGAP_Br7). These libraries contained 7985 “cancer” and “942” normal EST sequences.

### EST Mapping and *QSOX1* Gene Extension

To find out the extent of EST coverage of the of *QSOX1* mRNA, the long form of the reported *QSOX1* sequence (NM_002826) was searched against human EST (Expressed sequence tags) databases using BLAST (http://www.ncbi.nlm.nih.gov/BLAST). Following the identification of an EST fragment (DA998815) having a short 14 nucleotides overlap with the very 3′ end of the *QSOX1* cDNA and extending for over 500 bases downstream, another search for any ESTs downstream of the *QSOX1* gene was conducted using a fragment of human genomic DNA (AL390718, positions 147940 to 154450). A similar search was then performed using the non-redundant DNA database “nr/nt” to identify any cDNAs covering the region downstream of the reported exon 12 of the *QSOX1* gene. To identify putative alternatively spliced variants of *QSOX1*, its cDNA sequence (NM_002826) was searched against EST databases. Default BLAST search settings were used in all cases except the word size was set to 16, and no sequence filters were applied.

To identify possible correlations between *QSOX1* expression and the levels of two established breast cancer prognostic markers *ESR1* or *HER2*, 67 breast cancer SAGE expression libraries were analysed (last accessed 30/01/2011). Tag counts for short SAGE tag “AGCAGGTGCC” were used as a measure of *ESR1* expression, counts for short SAGE tag “AGGAAGGAAC” were used as a measure of *HER2* expression, and the counts for short SAGE tag “CTTGATTCCC” were used as a measure of *QSOX1* expression. The total number of tags were 63 (*ESR1*), 574 (*HER2*) and 355 for *QSOX1* across all 67 breast SAGE libraries (including 18 normal tissue derived, 10 cell line derived and 39 cancer tissue derived libraries).

### cDNA Preparation

T47D breast carcinoma cells were from European Collection of Cell Cultures (ECACC), Health Protection Agency Porton Down, Salisbury, SP4 0JG, UK. Total RNA was purified from cultured T47D cells using an Aurum Total RNA purification kit (Bio-Rad) and following the manufacturer’s protocol. Approximately 2x10^6^ cells were used and the RNA was finally eluted in 80 µl of elution buffer. cDNA was synthesized from 3 µl of the total RNA preparation, 100 pmol of Oligo-d(T)_21_ primer and using either iScript (Biorad) or BioScript (Bioline) kits and following the manufacturers’ recommendations. The synthesized cDNAs were diluted 10-fold with deionised water and stored at −20°C. Both cDNAs were tested by RT-PCR amplification of a short fragment of *GAPDH* cDNA using forward and reverse *GAPDH* gene specific primers, as detailed in Supplementary Table S2. cDNA obtained with the BioScript kit was used for RT-PCRs and cDNA produced using the iScript kit was used for 3′-RACE-PCR.

### RT-PCR

A Mastercycler gradient thermal cycler (Eppendorf) was used for RT-PCR amplifications. All the reactions were assembled using a REDTaq ReadyMix kit (Sigma-Aldrich). The amplification conditions included an initial denaturing step of 1 min at 96°C, followed by 30 cycles, each consisting of a 30 s denaturing step at 96°C, 30 s annealing step at (T_m_−7°C) and 1 min extension step at 72°C, and a final incubation of 5 min at 72°C. Primer sequences are listed in the Supplementary Table S2. All amplified cDNAs were analysed by electrophoresis in 1.5% agarose gels. All cDNAs were purified using a QIAquick PCR purification kit (QIAGEN) and their identity was confirmed by sequencing (GATC Biotech).

### 3′-RACE PCR

Two nested adapter oligonucleotides were devised to have identical annealing temperatures with the QSXO1-specific forward nested primers “105-Ext-F” and “106-Int-F”. One additional long adapter primer “Oligo-dT-21-Adap-0-R” containing Oligo(dT)_21_ was made based on the designed adapters “Adap-1-R” and “Adap-2-R”. Adapter primers were designed to have no significant sequence similarity with known human DNA sequences from the “nr/nt” database. All primer sequences are listed in Supplementary Table S2. Two rounds of 3′ RACE PCR amplification were performed using a Mastercycler gradient thermal cycler (Eppendorf). In the first round 2 µl of the Oligo-dT_(21)_- primed cDNA was amplified using a mixture of adapter primers “Oligo-dT-21-Adap-0-R” and “Adap-1-R” (at 1∶10 molar ratio) and one sequence specific primer “105-Ext-F”; and a REDTaq ReadyMix PCR kit (Sigma-Aldrich). The amplification procedure included one initial denaturing step for 1 min at 96°C, followed by 3 cycles, each consisting of a 30 s denaturing step at 96°C, 30 s annealing step at 65°C and 3 min extension step at 72°C, following by another 3 cycles, each consisting of a 30 s denaturing step at 96°C, 30 s annealing step at 50°C and 3 min extension step at 72°C, followed by 25 cycles of a 30 s denaturing step at 96°C, 30 s annealing step at 65°C and 3 min extension step at 72°C. Final extension was at 72°C for 5 min. The second round of 3′-RACE-PCR used primers “106-Int-F” and “Adap-2-R” and 0.2 µl of the first round of 3′-RACE-PCR reaction. The amplification conditions were: initial denaturing for 1 min at 96°C, followed by 30 cycles of a 30 s denaturing step at 96°C, 30 s annealing step at 56°C and 3 min extension step at 72°C. Final extension was at 72°C for 5 min. The amplified cDNA was analysed by electrophoresis in 2% agarose gels, purified using a QIAquick PCR purification kit (QIAGEN) and sequenced (GATC Biotech).

### Real Time PCR

Quantitative PCR was performed on an AB 7500 Fast Real Time system (Applied Biosystems, Warrington, UK) as previously described [45]. Cycle-cycle fluorescence changes in each sample were measured to generate a kinetic profile of DNA amplification over a 40-cycle PCR reaction. The cycle threshold number (*C*_T_) at which amplification entered the exponential phase was determined to indicate the amount of target RNA in each tissue sample, thus a lower *C*_T_ value indicates a larger quantity of starting RNA. The total RNA amounts in each sample were normalised relative to the endogenous level of 18S rRNA transcripts. Three primer sets were used (Supplementary Table S2). The primer and probe sequences were based on the database sequence of Homo sapiens *QSOX1* (Set 1) and on the newly identified 3′UTR extension of *QSOX1* transcript (Sets 2 and 3). All primers and probes were designed using Primer Express 1.0 Software (PE Applied Biosystems). A collection of RNA samples derived from normal and malignant mammary tissue was analysed [45]. These samples, obtained under informed consent, comprised 9 samples of normal mammary tissue acquired from breast reduction surgery and 41 surgically removed breast cancer samples graded according to the Nottingham prognostic index [46]. The patient group was aged between 42 and 88 at diagnosis, with 7 being pre-menopausal. 25 of the tumours were classified as ductal, 9 were lobular, and the remainder were various other pathological types.

## Supporting Information

Figure S1 **Scatter plot showing lack of correlation between** ***QSOX1*** **and ESR1 or HER2 expression.** Horizontal axis - SAGE expression data for ESR1 or HER2 expressed as Log of the reported SAGE counts. Vertical axis - *QSOX1* SAGE counts, Log scale. *QSOX1* vs. HER2 (filled circles), *QSOX1* vs ESR1 (open circles). The expression data show no significant correlation. The Pearson correlation coefficient calculated for libraries where both ESR1 and *QSOX1* were detected was −0.06, and −0.03 for HER2 and *QSOX1*.
(TIF)

Table S1 **Upregulated expression of 1q genes in breast ductal carcinoma (having significance factor 0.01<P≤0.05).** The upregulated genes with the significance factor P≤0.01 are listed in Table 1.
(DOC)

Table S2 **Nucleotide sequences of the primers used for all PCR amplifications.**
(DOC)
