# Supplementary figures and images for: Elevated Transcription of the Gene QSOX1 Encoding Quiescin Q6 Sulfhydryl Oxidase 1 in Breast Cancer
Source: PLoS One. 2013 Feb 27;8(2):e57327. doi: 10.1371/journal.pone.0057327 (PMC3583868; doi:10.1371/journal.pone.0057327)

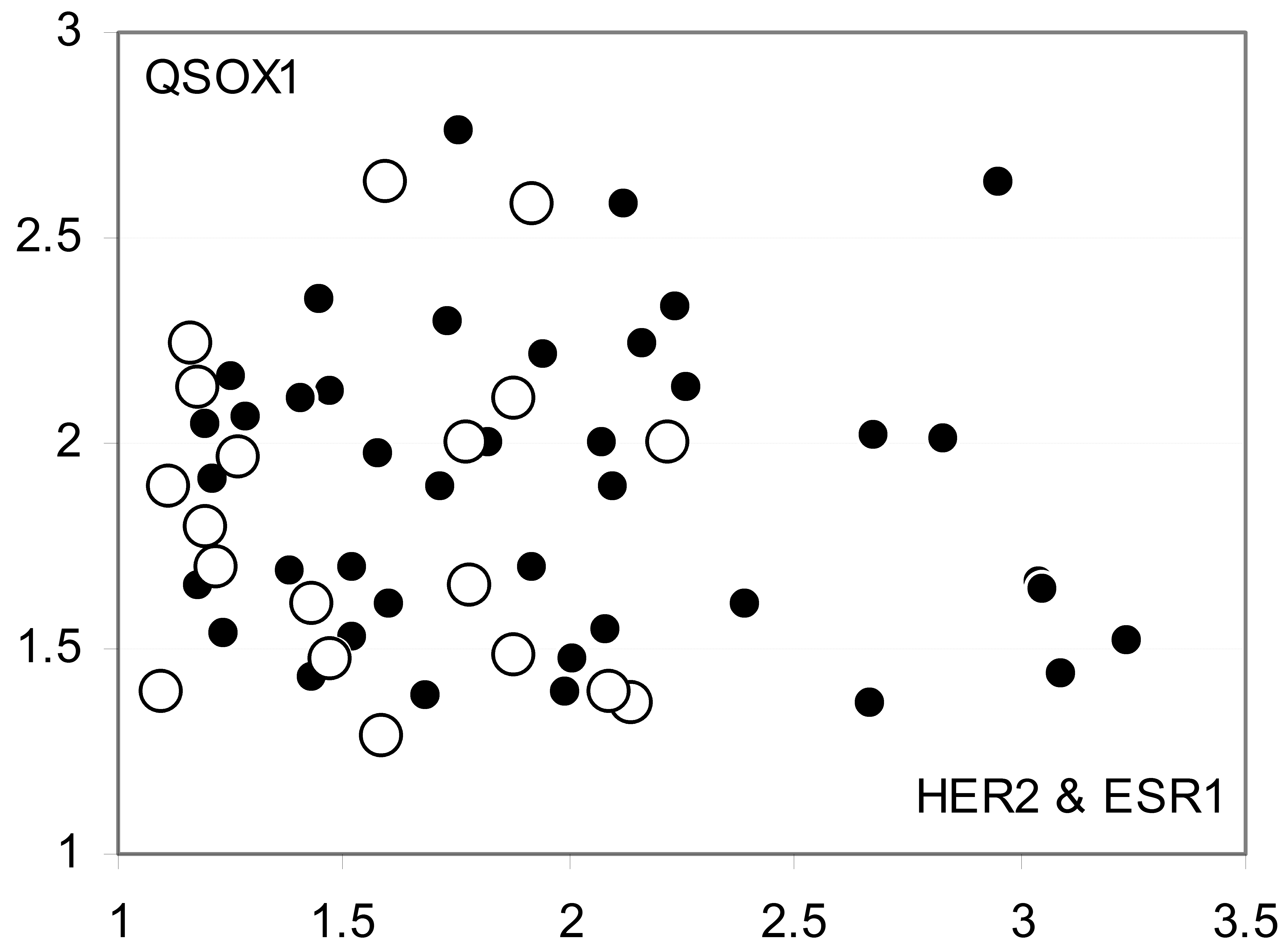

Supplement: Figure S1 — Scatter plot showing lack of correlation between QSOX1 and ESR1 or HER2 expression. Horizontal axis - SAGE expression data for ESR1 or HER2 expressed as Log of the reported SAGE counts. Vertical axis - QSOX1 SAGE counts, Log scale. QSOX1 vs. HER2 (filled circles), QSOX1 vs ESR1 (open circles). The expression data show no significant correlation. The Pearson correlation coefficient calculated for libraries where both ESR1 and QSOX1 were detected was −0.06, and −0.03 for HER2 and QSOX1. (TIF) [file pone.0057327.s001.tif]
