# Supplementary material for: Elevated Transcription of the Gene QSOX1 Encoding Quiescin Q6 Sulfhydryl Oxidase 1 in Breast Cancer
Source: PLoS One. 2013 Feb 27;8(2):e57327. doi: 10.1371/journal.pone.0057327 (PMC3583868; doi:10.1371/journal.pone.0057327)
Supplement: Table S1 — Upregulated expression of 1q genes in breast ductal carcinoma (having significance factor 0.01<P≤0.05). The upregulated genes with the significance factor P≤0.01 are listed in Table 1. (DOC) [file pone.0057327.s002.doc]

**Supplementary Table S1.** Upregulated expression of 1q genes in breast ductal carcinoma (having significance factor 0.01 < P ≤ 0.05).

| **Gene Symbol^1^** | **Gene Name** | **Chromosome position^2^** | **SAGE^3^ cancer (A)** | **SAGE^3^ normal (B)** | **NormalisedOdds A:B^4^** | **EST^5^ cancer (A)** | **EST^6^ normal (B)** |
| --- | --- | --- | --- | --- | --- | --- | --- |
| MEF2D | Myocyte enhancer factor 2D | **1q12-q23** | 8 | 0 | ∞ | **-** | **-** |
| EDEM3 | ER degradation enhancer, mannosidase alpha-like 3 | **1q24-q25** | 8 | 0 | ∞ | **-** | **-** |
| GNPAT | Glyceronephosphate O-acyltransferase | **1q42** | 8 | 0 | ∞ | **-** | **-** |
| KRTCAP2 | Keratinocyte associated protein 2 | **1q22** | 6 | 0 | ∞ | **+** | **-** |
| CAMSAP1L1 | Calmodulin regulated spectrin-associated protein family | **1q32.1** | 6 | 0 | ∞ | **-** | **-** |
| ZNF281 | Zinc finger protein 281 | **1q32.1** | 6 | 0 | ∞ | **-** | **-** |
| C1orf35 | Chromosome 1 open reading frame 35 | **1q42.13** | 6 | 0 | ∞ | **-** | **-** |
| ROBLD3 | Late endosomal/lysosomal adaptor, MAPK and MTOR activator | **1q22** | 10 | 1 | 5.61 | **-** | **-** |
| TUFT1 | Tuftelin 1 | **1q21** | 9 | 1 | 5.05 | **+** | **-** |
| EPHX1 | Epoxide hydrolase 1, microsomal (xenobiotic) | **1q42.1** | 9 | 1 | 5.05 | **+** | **-** |
| MUC1 | Mucin 1, cell surface associated | **1q21** | 11 | 2 | 3.09 | **-** | **-** |
| PRCC | Papillary renal cell carcinoma (translocation-associated) | **1q21.1** | 9 | 2 | 2.52 | **+** | **-** |
| HIST2H2AA4 | Histone cluster 2, H2aa4 | **1q21.2** | 13 | 3 | 2.43 | **-** | **-** |
| CENPL | Centromere protein L | **1q25.1** | 13 | 3 | 2.43 | **-** | **-** |
| JTB | Jumping translocation breakpoint | **1q21** | 11 | 3 | 2.06 | **+** | **+** |
| SRP9 | Signal recognition particle 9kDa | **1q42.12** | 12 | 4 | 1.68 | **+** | **-** |
| WDR26 | WD repeat domain 26 | **1q42.11-q42.12** | 13 | 5 | 1.46 | **+** | **-** |
| TPM3 | Tropomyosin 3 | **1q21.2** | 15 | 6 | 1.40 | **+** | **-** |
| TATDN3 | TatD DNase domain containing 3 | **1q32.3** | 15 | 6 | 1.40 | **-** | **-** |
| ATP1B1 | ATPase, Na+/K+ transporting, beta 1 polypeptide | **1q24** | 23 | 10 | 1.29 | **+** | **+** |
| UFC1 | Ubiquitin-fold modifier conjugating enzyme 1 | **1q23.3** | 16 | 7 | 1.28 | **+** | **-** |

^1^ The genes are listed in the order of degree of over-expression in cancer tissue. For genes with the significance factor is P < 0.01 see Table 1.

^2^ From Unigene (http://www.ncbi.nlm.nih.gov/UniGene)

^3^ Total number of short SAGE tags identified for each individual gene

^4^ The sequences Odds ratio is obtained by calculating normalised values of expression of each gene (total number of SAGE tags divided by the total number of tags in each library: 66,128 tags in the breast ductal carcinoma library and 50,512 tags in normal epithelium), and then calculating the ratio of these values (normalised expression in cancer over normalised expression in normal tissue). For the four top entries gene expression was not detected in normal tissues (SAGE only)

^5^ Based on ten non-normalised cDNA EST libraries from cancer breast tissues (not cell lines), totalling 11,161 sequences available

^6^ Based on two non-normalised cDNA EST libraries from normal breast tissues (not cell lines), totalling 1,485 sequences availabl
