# Supplementary material for: Elevated Transcription of the Gene QSOX1 Encoding Quiescin Q6 Sulfhydryl Oxidase 1 in Breast Cancer
Source: PLoS One. 2013 Feb 27;8(2):e57327. doi: 10.1371/journal.pone.0057327 (PMC3583868; doi:10.1371/journal.pone.0057327)
Supplement: Table S2 — Nucleotide sequences of the primers used for all PCR amplifications. (DOC) [file pone.0057327.s003.doc]

**Supplementary Table S2.** Nucleotide sequences of the primers used for PCR amplifications.

|  | **Oligonucleotide name** | **Oligonucleotide sequences (3' to 5')^1^** |
| --- | --- | --- |
| **Control GAPDH-specific primers** | GAP-F62 | AGCCAAAAGGGTCATCATCT |
|  | GAP-R62 | GCCTGCTTCACCACCTTC |
| **RT-PCR primers** | 23-F | TGGGTCAGGGTGGGGTCAAGTC |
|  | 36-R | TGACACATGGGGACAAGGGGATAA |
|  | 43-F | TTGGAGGGTGCCAGGT |
|  | 44-R | TGCCCCATAGCAGGAGTG |
|  | 45-F | GATGGCTGCTTTCACTTCC |
|  | 46-R | AGGCTTCTCCCTCACCAG |
|  | 47-F | GCCACTCGGTGGGGAGGAGT |
|  | 48-R | GCAAGCTCCAGCTCCCAGCC |
|  | 49-F | TTTCCCCAGGGCTCTCGGCA |
|  | 50-R | AGGGTCAAGGCTGGCTGGGT |
|  | 53-F | TAGAGGCTCTCCTGGCAAGAG |
|  | 54-R | GGTGGGAGTAAAGGGGATAGG |
|  | 63-F | CCACCCCCTGCCCCAATCCT |
|  | 64-R | GTGGACCTGGAGGCTGGGCT |
| **3'-RACE-PCR primers** | 105-Ext-F | TCATGTCGTGTCGTCTCCTGTCTTG |
|  | 106-Int-F | TGCCACTGGCCAGAGTT |
|  | Oligo-dT-21-Adap-0-R | GGCGCCCTAGATGACGTGAGCGGTTTTTTTTTTTTTTTTTTTTT |
|  | Adap-1-R | CGATCGGGCGCCCTAGATG |
|  | Adap-2-R | CTAGATGACGTGAGCGGTTT |
| **Real time qPCR primers** | Set1 TaqMan Probe ^2^ | 6FAM - CCCAGACCACAGTTGCACCAACCAC - TAMRA |
|  | Set1F ^2^ | TCCCCGTGCTCATGGAAT |
|  | Set1R ^2^ | GCGATCTGCCAATTTCCAAA |
|  | Set2 TaqMan Probe ^3^ | 6FAM - CTGCACCCAAACCCCATGGCC - TAMRA |
|  | Set2F ^3^ | CCACAGACTGCCCATAGAACTG |
|  | Set2R ^3^ | GCCTGTCTCCGTGCATGAA |
|  | Set3 TaqMan Probe ^4^ | 6FAM - AGACGAGTAGGTGTTAGC - MGB |
|  | Set3F ^4^ | GACAGTGGCATGGAGCTTTGA |
|  | Set3R ^4^ | CCCCGTTCCTCCTTATTTCC |

^1^ 6FAM denotes 6-carboxyfluorescein, MGB denotes dihydrocyclopyrroloindole tripeptide minor groove binder. TAMRA denotes tetramethylrhodamine.

*^2^ Set1 is based on Exon 7 of QSOX1*

*^3^ Set2 is based on newly identified expended 3'UTR (pos. 148085 to 148149 of AL390718)*

*^4^ Set3 is based on newly identified expended 3'UTR (pos. 149393 to 149454 of AL390718)*
